# Differential actions of PPAR-α and PPAR-β/δ on beige adipocyte formation: A study in the subcutaneous white adipose tissue of obese male mice

**DOI:** 10.1371/journal.pone.0191365

**Published:** 2018-01-19

**Authors:** Tamiris Lima Rachid, Flavia Maria Silva-Veiga, Francielle Graus-Nunes, Isabele Bringhenti, Carlos Alberto Mandarim-de-Lacerda, Vanessa Souza-Mello

**Affiliations:** Laboratory of Morphometry, Metabolism, and Cardiovascular Diseases, Biomedical Center, Institute of Biology, State University of Rio de Janeiro, Rio de Janeiro, Brazil; Tokyo University of Agriculture, JAPAN

## Abstract

**Background and aims:**

Obesity compromises adipocyte physiology. PPARs are essential to adipocyte plasticity, but its isolated role in the browning phenomenon is not clear. This study aimed to examine whether activation of PPAR-α or PPAR-β/δ could induce beige cell depots in the subcutaneous white adipose tissue of diet-induced obese mice.

**Material and methods:**

Sixty animals were randomly assigned to receive a control diet (C, 10% lipids) or a high-fat diet (HF, 50% lipids) for ten weeks. Then each group was re-divided to begin the treatments that lasted 4 weeks, totalizing six groups: C, C-α (C plus PPAR-α agonist, 2.5 mg/kg BM), C-β (C plus PPAR-β/δ agonist, 1 mg/kg BM), HF, HF-α (HF plus PPAR-α agonist), HF-β (HF plus PPAR-β/δ agonist).

**Results:**

HF animals presented with overweight, glucose intolerance and subcutaneous white adipocyte hypertrophy. Both treatments significantly attenuated these parameters. Browning, verified by UCP1 positive beige cells and enhanced body temperature, was just observed in PPAR-α treated groups. PPAR-α agonism also elicited an enhanced gene expression of the thermogenesis effector UCP1, the beige-selective gene TMEM26 and the PRDM16, an essential gene for brown-like phenotype maintenance in the beige adipocytes when compared to their counterparts. The enhanced CIDEA and the reduced UCP1 gene levels might justify the white phenotype predominance after the treatment with the PPAR-β/δ agonist.

**Conclusions:**

This work provides evidence that the PPAR-β/δ agonist ameliorated metabolic disorders through enhanced beta-oxidation and better tolerance to glucose, whereas the PPAR-α agonism was confirmed as a promising therapeutic target for treating metabolic diseases via beige cell induction and enhanced thermogenesis.

## Introduction

Obesity has a growing prevalence in both developed and developing countries [1]. White adipose tissue (WAT) remodeling in obesity results in inflammation, insulin resistance, enhanced lipolysis, ectopic lipid accumulation and reduced energy expenditure (EE) [2]. WAT is currently considered as a metabolically active organ of an endocrine nature [3]. The secretion of adipokines and their roles in the regulation of energy homeostasis, inflammatory response, and glucose tolerance are altered in obese individuals [4].

FNDC5/Irisin, an adipokine secreted mainly by the subcutaneous WAT (sWAT) [5], TMEM26 (a beige-selective gene), and PRDM16 (essential to maintain the brown-like phenotype) are markers for the the formation of beige adipocytes [6, 7]. Considering that the brown adipose tissue (BAT) of adult humans is composed of beige adipocytes, experimental approaches capable of inducing the browning of sWAT can configure translational strategies to tackle obesity in humans by enhancing thermogenesis [8].

Adaptive thermogenesis is dependent on uncoupling protein 1 (UCP1) and produces heat from chemical energy (carbon atoms ingested in the diet), enhancing EE and combating hypothermia and obesity [9, 10]. UCP1 functions as an alternative channel that allows protons to return to the mitochondrial matrix from the intermembrane space, without producing energy as ATP, and, therefore, uncoupling the electron transport and phosphorylation reactions [11].

Previous data showed that elevated FNDC5/irisin, peroxisome proliferator-activated receptor (PPAR)-α and PPAR-β/δ gene levels, after the administration of fenofibrate (PPAR-α agonist) to obese mice, led to UCP1 positive beige adipocytes formation, which expressed typical BAT genes (PRDM16 and BMP8b) in the sWAT, indicating enhanced thermogenic activity [12].

Despite the evidence that PPAR-α activation by fenofibrate stimulates browning, there appears to be also a contribution of the PPAR-β/δ isoform to this outcome. Hence, this work aimed to evaluate the differential effects of a purified PPAR-α agonist and a purified PPAR-β/δ agonist on the white-brown sWAT plasticity and the potential for adaptive thermogenesis in a diet-induced obesity murine model.

## Material and methods

### Animals and diet

Male C57BL/6 mice (3 months old) were maintained on a 12 h/12 h dark/light cycle with controlled humidity (60 ± 10%) and temperature (21 ± 2° C) in pathogen-free cages with free access to food and water. This study was in accordance with the conventional guidelines for experimentation with animals (National Institutes of Health Publication No. 85–23, revised in 1996) and all procedures were approved by the Animal Ethics Committee of State University of Rio de Janeiro (Protocol Number CEUA/034/2016).

Thirty mice were submitted to a high-fat diet (HF: 14% of energy as protein, 50% as fat and 36% as carbohydrates, total energy 21 KJ/g). Simultaneously, others thirty mice received a control diet (C: 14% of energy as protein, 10% as fat, and 76% as carbohydrates, total energy 15 KJ/g). Both diets followed the recommendations by the AIN-93M for rodents, and this protocol lasted ten weeks [13]. Subsequently, the treatment started and groups C and HF were separated into six new groups (n = 10 each group) as follows: a) C group–untreated, fed the C diet throughout the experiment; b) C-α group–C diet treated with PPAR-α agonist (WY-14643, 2.5mg/kg); c) C-β group–C diet treated with PPAR-β/δ agonist (GW0742, 1mg/Kg); d) HF group–untreated, fed the HF diet throughout the experiment; e) HF-α group–HF diet treated with PPAR-α agonist (WY-14643, 2.5mg/kg); f) HF-β group–HF diet treated with PPAR-β/δ agonist (GW0742, 1mg/Kg). The treatments lasted four weeks, and the PPAR agonists (Sigma-Aldrich, St. Louis, MO, USA) were mixed into the diet.

The diets were manufactured by PragSolucoes (Jau, Sao Paulo, Brazil). Food intake was measured daily, and body mass (BM) was measured weekly. Energy intake (in KJ) was obtained as the product of food consumption and the energy content of the diet.

### Oral glucose tolerance test (OGTT)

The OGTT was performed after 6 h of food deprivation in the last week of the experiment. Glucose (1.0 g/kg) was administered by orogastric gavage, and blood was collected by milking the tip of the tail at 0, 15, 30, 60 and 120 min after glucose administration, and blood glucose was measured with a glucometer (Accu-Chek, Roche, São Paulo, SP, Brasil). The area under the curve (AUC) was calculated using the trapezoid rule (GraphPad Prism v7.03 for Windows, GraphPad Software, La Jolla, CA, USA). The blood glucose level at time 0 was considered the fasting glucose level.

### Thermography

Body temperature was measured by thermographic analysis in conscious animals at room temperature using an infrared camera FLIR C2 (FLIR Systems, Wilsonville, Oregon, USA).

### Sacrifice and tissue extraction

After the 14-week protocol, animals were fasted overnight and then deeply anesthetized (intraperitoneal sodium pentobarbital, 150 mg/g). Blood samples were obtained by cardiac puncture. The plasma was separated by centrifugation at room temperature (120 g for 20 min) and stored at -20°C until assay.

The subcutaneous inguinal fat pad, located in the lower part of the rib cage and the mid-thigh, was considered as the sWAT. All fat pads (epididymal, retroperitoneal and inguinal) were carefully dissected and weighed for calculating the adiposity index determined as the ratio between the sum of all fat pads masses divided by the total BM, represented as a percentage. sWAT was also prepared for routine histopathology or frozen to RT-qPCR analyses.

### Plasma analysis

Plasma concentrations of FNDC5/irisin and insulin were evaluated in duplicate with commercially available enzyme-linked immunosorbent assay kits (Mouse FNDC5/ Irisin ELISA kit Cat. # SEN576Mu, Uscn Life Science Inc., Houston, Texas, USA and Rat/Mouse Insulin ELISA kit Cat. #EZRMI-13K, Millipore, Missouri, USA), using the Fluostar Omega equipment (BMG LABTECH GmbH, Germany).

### Light microscopy and adipocyte stereology

Formalin-fixed sWAT samples were embedded in Paraplast Plus (Sigma-Aldrich, St. Louis, MO, USA) following routine procedures, cut with five micrometers of thickness and stained with hematoxylin and eosin. Ten non-consecutive random microscopic fields were analyzed per animal on a light DMRBE Leica microscope (Leica Microsystems GmbH, Wetzlar, Germany), using an Infinity 1-5c camera (Lumenera Co., Ottawa, ON, Canada). The average cross-sectional area of the adipocytes was estimated by stereology as the ratio between the volume density of adipocytes and twice the numerical density of adipocyte per area [14].

### Immunofluorescence

After sWAT sample deparaffinization, antigen retrieval and nonspecific binding blockade were performed as previously described [12]. Sections were incubated with anti-UCP1 (anti-goat, SC-6529, Santa Cruz Biotechnology) primary antibody and secondary anti-goat Alexa Fluor 546 conjugated antibodies were used. Slides were mounted with Slow Fade (Invitrogen, Molecular Probes, Carlsbad, CA, USA) to maintain fluorescence. Control sections were obtained after the omission of the primary antibodies. A fluorescence microscope (BX51, Olympus America Inc., Florida, USA) with a DP71 camera was used to assess the slides.

### RT-qPCR

Total RNA was extracted from 50 mg sWAT using Trizol reagent (Invitrogen, CA, USA). RNA quantity was determined using Nanovue (GE Life Sciences) spectroscopy, and 1 mg RNA was treated with DNAse I (Invitrogen). First-strand cDNA synthesis was performed using Oligo (dT) primers for mRNA and Superscript III reverse-transcriptase (both Invitrogen). Quantitative real-time PCR (RT-qPCR) was performed using a StepOne plus cycler and the SYBR Green mix (Invitrogen). Primers were designed using Primer3web online software version 4.0.0 and are indicated in Table 1. The beta-actin gene was used as an endogenous control to normalize selected gene expression. Efficiencies of RT-qPCR for the target gene and the endogenous control were approximately equal and were calculated from a cDNA dilution series. Real-time PCR reactions were conducted as previously detailed [12]. Negative controls consisted of wells in which cDNA was substituted for deionized water. The relative mRNA expression ratio (RQ) was calculated using the 2^-∆∆ct^ equation in which 2∆CT expresses the difference between the number of cycles (CT) of the target genes and the endogenous control.

**Table 1:** Detailed forward and reverse primer sequences of RT-qPCR.

Primers		
Name	Forward	Reverse
*PPAR-α*	TCGGACTCGGTCTTCTTGAT	TCTTCCCAAAGCTCCTTCAA
*PPAR-β/δ*	TGGAGCTCGATGACAGTGAC	GGTTGACCTGCAGATGGAAT
*PGC1- α*	GTCAACAGCAAAAGCCACAA	GTGTGAGGAGGGTCATCGTT
*NFR1*	GTTGGTACAGGGGCAACAGT	GTAACGTGGCCCAGTTTTGT
*TFAM*	GAAGAACGCATGGAGGAGAG	TTCTGGGGAGAGTTGCAGTT
*BMP-8b*	CTATGCAGGCCCTGGTACAT	AGGCCTGGACTACCATGTTG
*PRDM16*	AGGGCAAGAACCATTACACG	GGAGGGTTTTGTCTTGTCCA
*FNDC5/Irisin*	GGTGCTGATCATTGTTGTGG	CGCTCTTGGTTTTCTCCTTG
*UCP1*	TCTCAGCCGGCTTAATGACT	TGCATTCTGACCTTCACGAC
*β3-AR*	ACAGGAATGCCACTCCAATC	TTAGCCACAACGAACACTCG
*PPAR-γ*	ACGATCTGCCTGAGGTCTGT	CATCGAGGACATCCAAGACA
*TMEM-26*	GCTCACCCTCAAGTTCAAGC	TGCATTTCAAGAAGCCACAG
*CIDEA*	CTCGGCTGTCTCAATGTCAA	GGAACTGTCCCGTCATCTGT
*CPT-1b*	GGCTGCCGTGGGACATT	TGCCTTGGCTACTTGGTACGA
*Beta-actin*	CTCCGGCATGTGCAA	CCCACCATCACACCCT

**Abbreviations**: Peroxisome Proliferator Activated Receptors (PPAR), PPAR-*alpha* (PPAR-α), PPAR-*beta/delta* (PPAR-β/δ), PPAR-gamma (PPAR-γ), peroxisome proliferator-activated receptor gamma coactivator 1-alpha (PGC1- α), nuclear respiratory factor 1 (NFR1), mitochondrial transcription factor A (TFAM), bone morphogenetic protein-8b (BMP-8b), PR domain containing 16 (PRDM16), fibronectin type III domain-containing protein 5 (FNDC5)/irisin, uncoupling protein-1 (UCP1), β3-AR (beta 3 –adrenergic receptor), transmembrane protein 26 (TMEM26), cell death-inducing DFFA-like effector a (CIDEA) and carnitine palmitoyltransferase I b (CPT-1b).

### Data analysis

Values are expressed as the means and standard deviation (SD). We confirmed the normal distribution and homoscedasticity of variances, then we compared the groups in the first ten weeks of the experiment with t-test, otherwise by analysis of variance (ANOVA) followed by the Holm-Sidak post-hoc test. We tested the possible interactions between diet and treatment with a two-way ANOVA. In all cases, P-value<0.05 was accepted as statistically significant (GraphPad Prism v7.03 for Windows, GraphPad Software, La Jolla California USA). Considering the limited blood and sWAT sample from the mouse strain used, the minimum sample number of five animals per group was considered to carry out some analysis to guarantee the significance level adopted [15].

## Results

### PPAR-α and PPAR-β/δ agonists reduce body mass, without changing energy intake of obese mice

**Fig 1:**
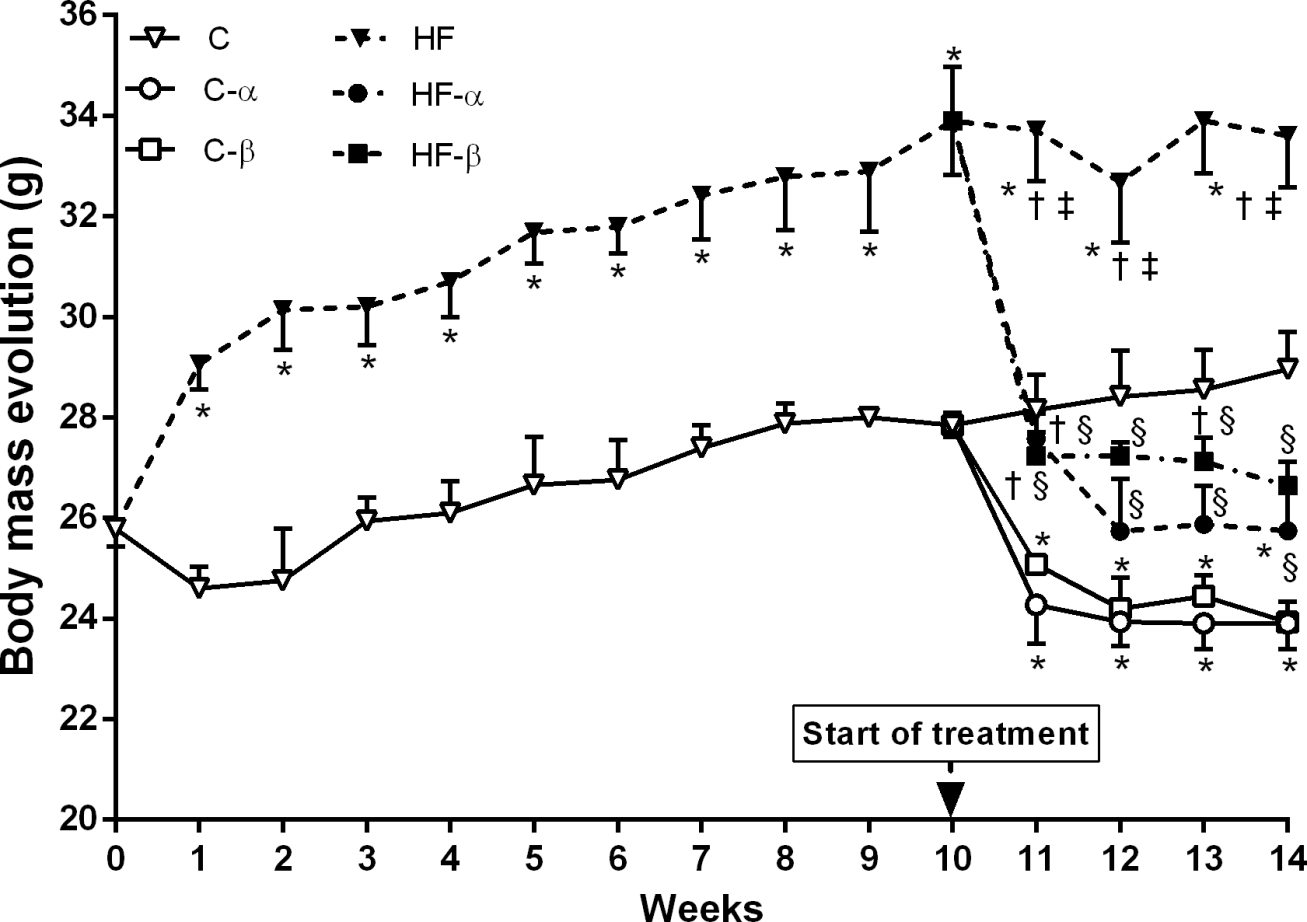
Body mass evolution. Weeks 1–10 correspond to the obesity induction protocol, and weeks 10–14 correspond to the treatment time. Values are the means ± SD, n = 10. Differences between the groups are indicated: P<0.05 when compared with the C group [*], C-α group [†], C-β group [‡] and HF group [§] (one-way ANOVA and post-hoc test of Holm-Sidak). Groups: C (control group), α (PPAR-α agonist WY14643), β (PPAR-β/δ agonist GW0742), HF (High-fat diet group).

The C and HF groups began the experiment without a difference in their initial BM (week 0). After one week of dietary administration, the HF group had a higher BM than the C group (+18%, P = 0.0084), which continued to increase until ten weeks of diet intake (+22%; P = 0.0124). At the end of the treatment, the HF-α and HF-β groups were lighter (-24% and -21%, P <0.0001 respectively) than the HF group. Similarly, the C-α and C-β groups showed a significant reduction of BM compared to the C group at the end of the experiment (-17% both groups, P <0.001, Fig 1). Two-way ANOVA revealed that the treatment with PPAR-α and PPAR-β/δ agonists influenced the final BM greatly, accounting for 65% and 62% of its total variance (P<0.0001). The diet also influenced this parameter, albeit with low intensity (P = 0.002).

The average weekly food intake in grams was not significantly different between the six groups over the fourteen-week experimental period (Table 2). Energy intake was more significant in the HF, HF-α and HF-β groups than in their counterparts (+36%, +28%, +50% and P<0.05, respectively), as indicated by calculating the energy provided by the diets based on their energy density (Table 2). Only the diet influenced the energy intake significantly (Two-way ANOVA, P<0.0001).

**Table 2:** Food behavior (during treatment), carbohydrate metabolism, body temperature, hormones and adipocyte morphology.

Data	C	C-α	C-β	HF	HF-α	HF-β
Food intake (g/day/animal)	2.37±0.04	2.32±0.02	2.72±0.19	2.46±0.03	2.31±0.05	2.705±0.16
Energy intake (KJ/day/animal)	37.74±0.65	36.95±0.37	43.35±3.012	51.41±0.55*^†^	48.28±1.26*^†^	56.5345±4.13* ^‡^
Fasting glucose (mmol/L)	7.14±0.45	6.05±1.01	6.20±1.15	9.82±0.78* ^† ‡^	6.16±1.01^§^	8.36±0.66^† ‡ ¤^
Temperature (°C)	33.24±0.69	36.62±1.51*	34.04±0.23^†^	34.32±0.60^†^	36.46±0.53* ^‡ §^	35.70±0.90* ^‡^
Insulin (pg/mL)	756.5±199.30	658.00±171	481.10±194	1326.00±253.40* ^† ‡^	874.50±209.10^‡ §^	471.60±151.60 ^§ ¤^
Irisin (ng/mL)	43.56±1.36	84.69±18.35*	37.01±15.19^†^	28.27±5.12^†^	100.40±8.90 ^‡ §^	34.99±12.43^† ¤^
Adiposity index (%)	3.49±0.15	2.48±0.63*	2.42±0.52*	4.48±0.23* ^† ‡^	3.16±0.39^§^	4.31±0.56^† ‡ §^
Adipocyte average cross-sectional area (μm^2^)	98.61±22.06	27.80±11.73*	22.23±8.05*	262.80±60.80* ^† ‡^	83.18±17.76^§^	40.06±13.29 ^§^

Values are shown as mean ± SD, n = 5 per group. P<0.05 when compared with the C group [*], C-α group [†], C-β group [‡], HF group [§] and HF-α [¤] (one-way ANOVA and post-hoc Holm-Sidak test).

### PPAR-α and PPAR-β/δ agonists tackle glucose intolerance in mice fed the high-fat diet

The HF group presented with a higher fasting glucose than the C group (+38%, P = 0.0007). PPAR-α agonist treatment could reduce the glycemia in the HF-α group when compared to the HF group (-37%, P<0.0001), completely rescuing this parameter as the HF-α animals did not show the statistical difference when compared to the C group (Table 2). Two-way ANOVA confirmed these observations as the treatment with the PPAR-α agonist accounted for 49% of fasting glucose’s total variance (P<0.0001), besides interacting significantly with the diet to determine this parameter (P = 0.0036).

**Fig 2:**
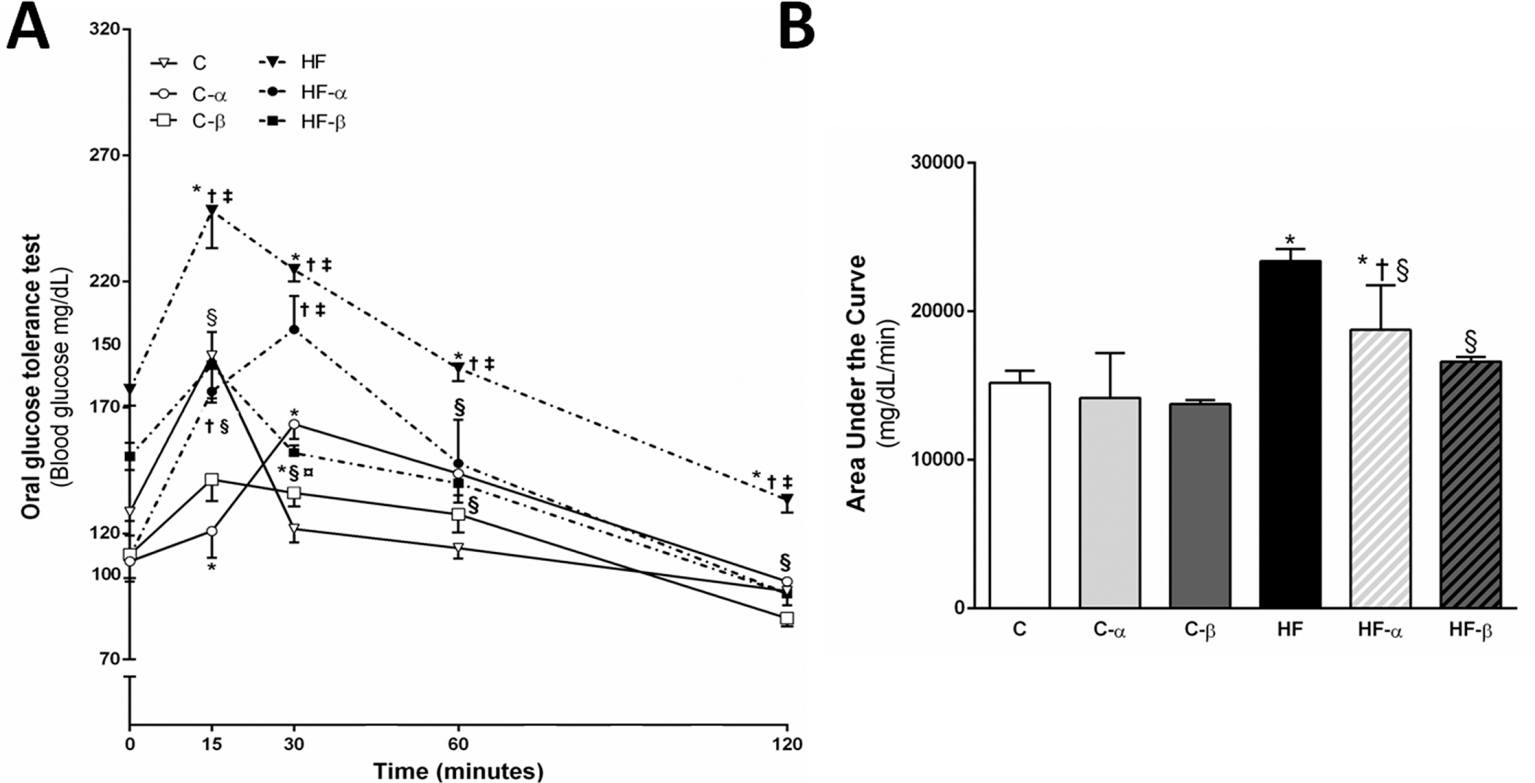
**Oral glucose tolerance test curve (A), and the area under the curve (B).** Values are the means ± SD, n = 5. Differences between the groups are indicated: P<0.05 when compared with the C group [*], C-α group [†], C-β group [‡], HF group [§] and HF-α [¤] (one-way ANOVA and post-hoc test of Holm-Sidak). Groups: C (control group), α (PPAR-α agonist WY14643), β (PPAR-β/δ agonist GW0742), HF (High-fat diet group).

The OGTT results were in agreement with the fasting glucose result as the HF group presented with a higher AUC, indicating glucose intolerance, compared with the C and C- α groups (+54% and +65%; P<0.0001). The treatment with PPAR-α and PPAR-β/δ agonists normalized the glucose tolerance caused by the HF diet (-20%, P<0.05 and -29%, P<0.001). Of note, HF-α and HF-β groups did not differ statistically from the C group regarding AUC values, highlighting an insulin-sensitizing effect of both treatments. OGTT results are found in Fig 2.

The two-way ANOVA exhibited different results for the HF-α and HF-β groups. In the former, only diet (P<0.0001) and treatment (P = 0.0114) as a single stimulus influenced the AUC values significantly. Conversely, in the latter, apart from a significant influence of diet (P<0.0001) and treatment (P<0.0001) independently, there was also a significant interaction between these factors to determine the AUC results (P<0.0001).

### PPAR-α agonist, but not PPAR-β/δ, increases body temperature of obese mice

**Fig 3:**
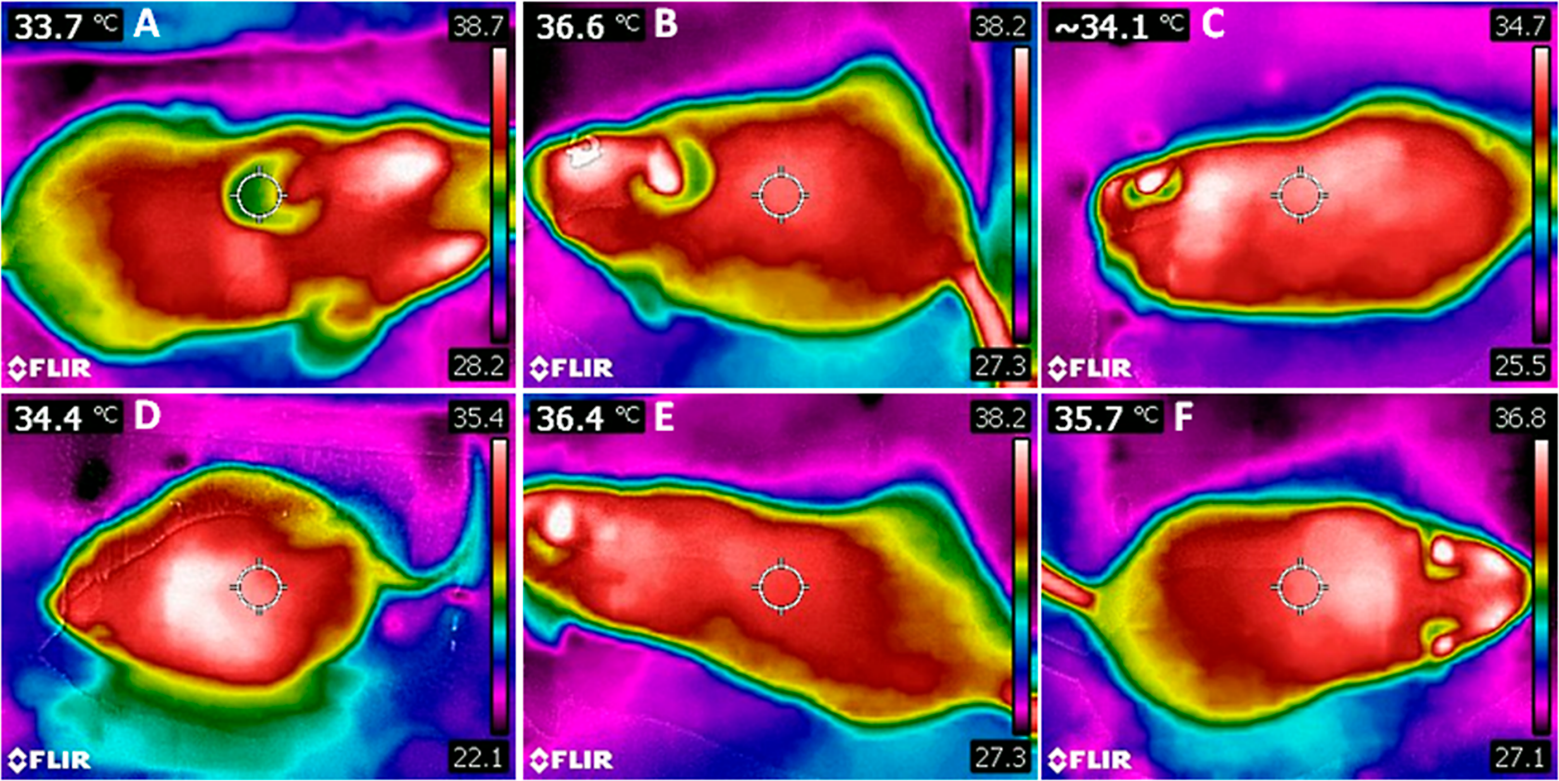
**Dorsal view of infrared thermography**: C group (A), C-α group (B), C-β group (C), HF group (D), HF-α group (E) and HF-β group (F). Groups: C (control group), α (PPAR-α agonist WY14643), β (PPAR-β/δ agonist GW0742), HF (High-fat diet group).

PPAR-α treatment yielded a higher body temperature in C-α and HF-α than in C and HF groups (+10%, P<0.0001; and +6%, P<0.005). Treatment with PPAR-β/δ did not alter body temperature. These data were illustrated in Fig 3, and the detailed information is found in Table 2. PPAR-α treatment accounted for 70% of body temperature’s total variance, exerting a significant influence on this parameter (Two-way ANOVA, P<0.0001). In contrast, the PPAR-β/δ agonist accounted for only 26% of its total variance (P = 0.0018), which was not enough to change this parameter.

### PPAR-α and PPAR-β/δ agonists counter hyperinsulinemia, but only PPAR-α agonist raises FNDC5/irisin levels in obese mice

The HF group showed higher insulin levels than the C group (+75%, P<0.05) and both treatments countered the hyperinsulinemia caused by chronic HF intake (-34% for HF-α and -64% for HF-β, P<0.05). Two-way ANOVA revealed that diet and PPAR-α treatment, as single stimulus, influenced significantly the insulinemia (P = 0.0007 and P = 0.0099, respectively). Conversely, PPAR-β/δ agonist exerted a single influence on plasma insulin levels (P<0.0001), besides interacting significantly with the diet to affect this parameter (P = 0.0036).

FNDC5/Irisin levels were more significant in C-α group than in the C group (+94%, P<0.005) as well as in the HF-α when compared to HF group (+255%, P<0.0001). The treatment with PPAR-β/δ agonist had no significant effect on the levels of this adipokine. These results are shown in Table 2. Importantly, only the PPAR-α treatment influenced FNDC5/irisin levels, accounting for 84% of its total variance (Two-way ANOVA, P<0.0001). Moreover, diet and PPAR-α agonist interacted affecting the results (Two-way ANOVA, P = 0.0047).

### PPAR-α and PPAR-β/δ agonists reduces the adiposity index of obese mice

The adiposity index was reduced after PPAR-α and PPAR-β/δ treatment in the C-α and the C-β groups when compared to the C group (-29% and -31%, P<0.05 respectively). In agreement with the increased energy intake, the HF group had a higher adiposity index than the C group (+28%, P<0.05). Both treatments tackled this metabolic impairment as HF-α and HF-β groups had a reduced adiposity index when compared to the untreated HF group (-29% and -23%, P<0.05 respectively). These results are demonstrated in Table 2.

Two-way ANOVA showed that diet and both treatments influenced the adiposity index independently (P<0.0001). It is noteworthy that PPAR-α treatment accounted for 53%, whereas PPAR-β/δ treatment accounted for 42% of its total variance.

### Both treatments reduce adipocyte size, but only PPAR-α agonist induces the formation of beige adipocytes

White adipocytes from the HF group were considerably enlarged when compared with the C, C-α and C-β groups (+166%, +845% and +1.082%, P<0.0001 respectively). PPAR-α agonist yielded a smaller adipocyte average cross-sectional area in the C-α group than in the C group (-89%, P = 0.0226), and in the HF-α group than in the HF group (-68%, P<0.0001). PPAR-β/δ agonist was also able to ameliorate this deleterious effect by reducing adipocyte average cross-sectional area significantly in the C-β and HF-β groups in comparison with C and HF (-78%, P = 0.0120 and -85%, P<0.0001, Table 2). Two-way ANOVA showed that diet (P<0.0001) and both treatments (P<0.0001), as single factors, influenced the adipocyte cross-sectional area, besides interacting significantly to determine this parameter (P = 0.0002).

**Fig 4:**
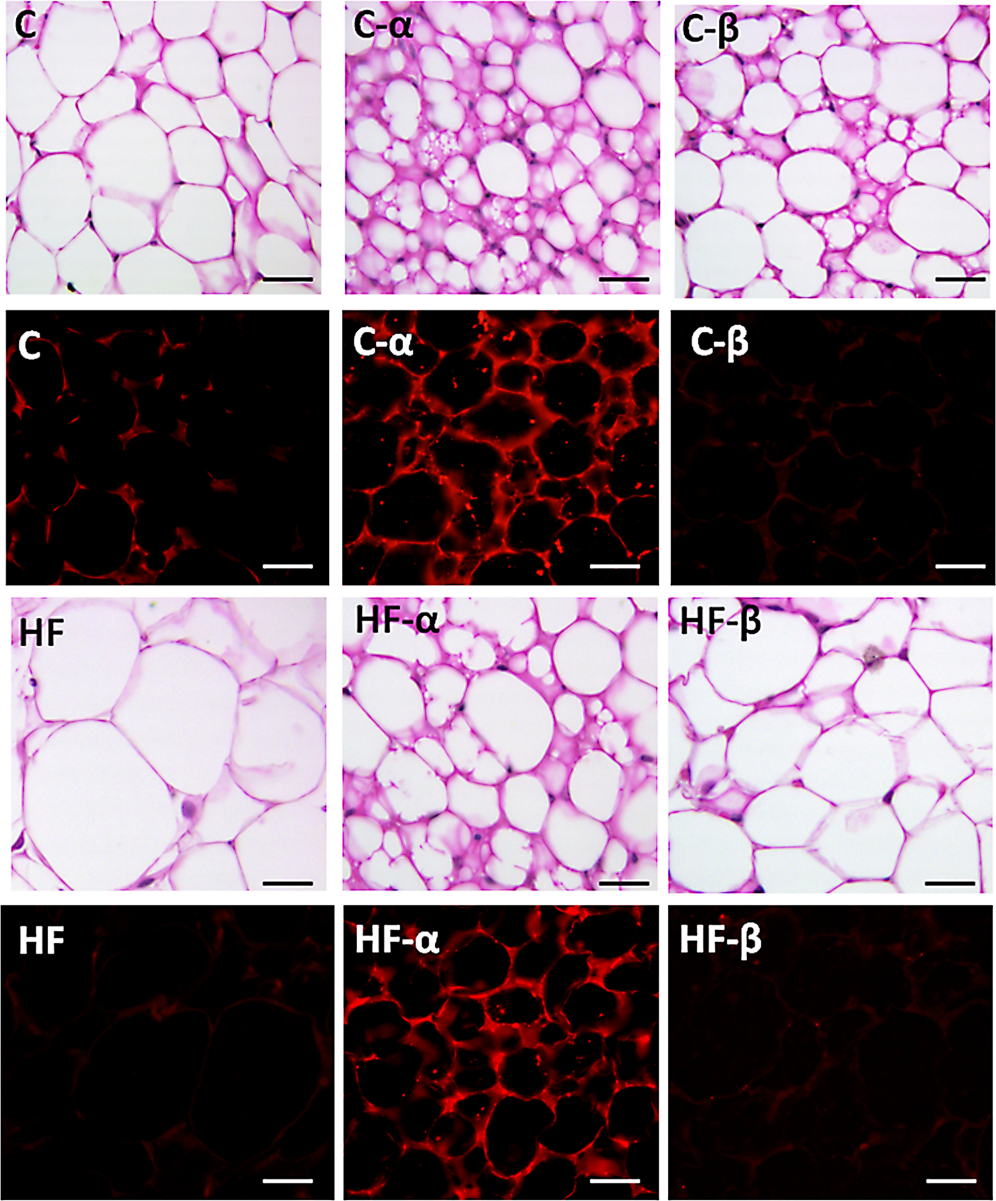
sWAT hematoxylin and eosin staining and immunofluorescence for UCP-1 (red label). The line with hematoxylin and eosin staining is followed by the line with immunofluorescence for UCP1. All images were obtained with the same magnification (calibration bar = 50 μm). Groups: C (control group), α (PPAR-α agonist WY14643), β (PPAR-β/δ agonist GW0742), HF (High-fat diet group).

The photomicrographs in Fig 4 demonstrate unilocular sWAT adipocytes in the untreated groups (C and HF). The presence of beige cell clusters in the PPAR-α treated groups (C-α and HF-α) is noticeable and suggests the phenomenon known as "browning." Both, C-α and HF-α groups presented with areas of multilocular adipose tissue within the specific unilocular WAT cells, which have been called beige or brite (“brown in white”) adipose tissue. This phenomenon was not observed in the groups treated with PPAR-β/δ agonist.

### PPAR-α treated animals, but not PPAR- β/δ treated ones, show positive UCP1 beige adipocytes in the subcutaneous white adipose tissue

The “browning” phenomenon and the presence of beige adipocytes, initially observed by the histological images, were confirmed in the C-α and HF-α groups by positive immunofluorescence UCP1 staining in the sWAT of these groups. Conversely, subcutaneous white adipocytes from untreated C, HF, and PPAR-β/δ treated groups did not show this pattern of immunoreactivity as UCP1 is closely related to thermogenesis and is markedly expressed in brown adipocytes. These data are shown in Fig 4.

### PPAR-α treated animals show enhanced expression of beige-selective and thermogenic genes, while PPAR-β/δ treated animals showed favored beta-oxidation

**Fig 5:**
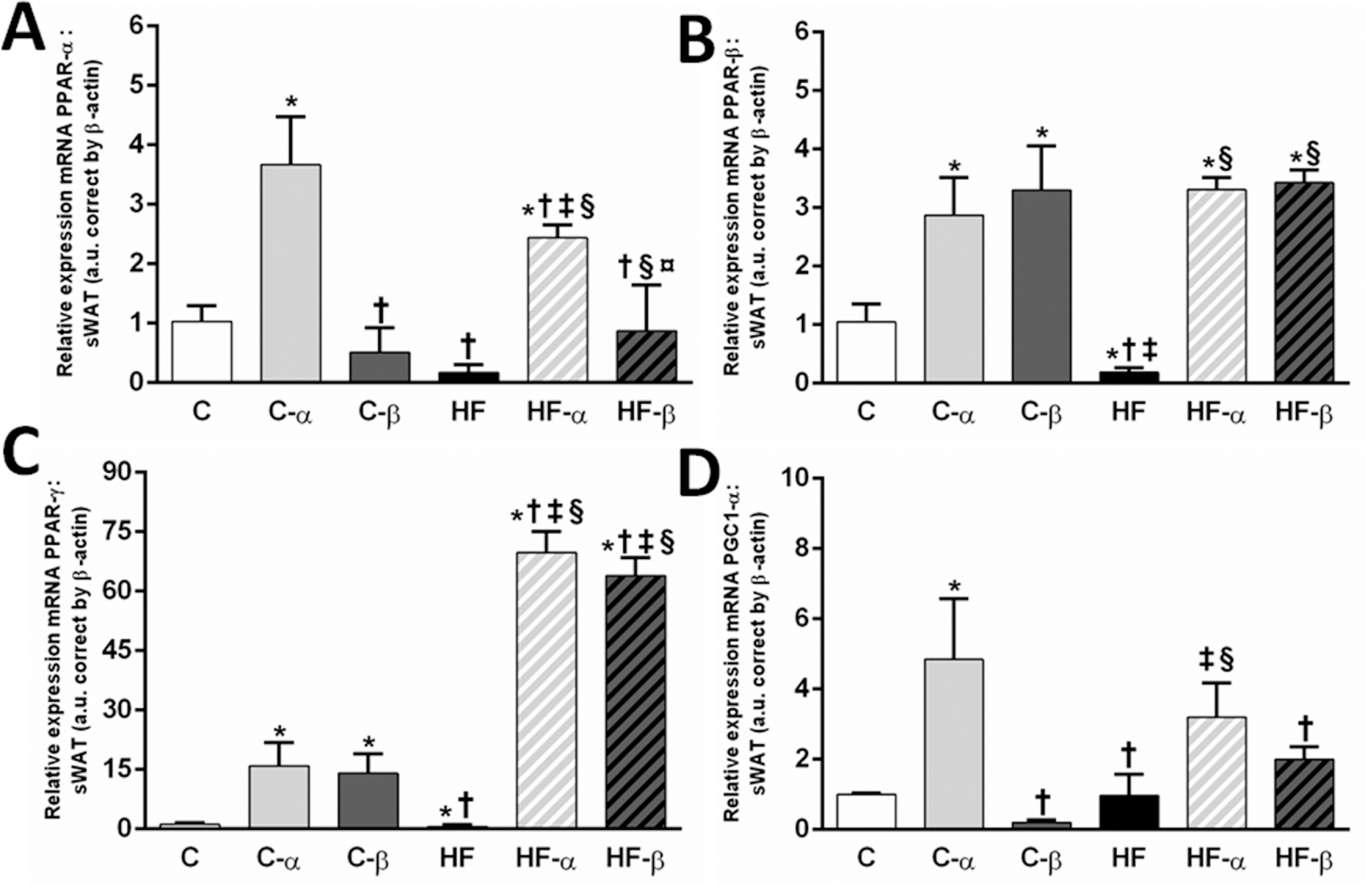
**sWAT gene expressions of *PPAR-α* (A), *PPAR-β/δ* (B), PPAR-γ (C) and *PGC1-α* (D).** Beta-actin was used as an internal control to normalize selected gene expression. Values are the means ± SD, n = 5. Differences between the groups are indicated: P<0.05 when compared with the C group [*], C-α group [†], C-β group [‡], HF group [§] and HF-α [¤] (one-way ANOVA and post-hoc test of Holm-Sidak). Groups: C (control group), α (PPAR-α agonist WY14643), β (PPAR-β/δ agonist GW0742), HF (High-fat diet group).

Treatment with the PPAR-β/δ agonist did not alter *PPAR-α* gene levels. In contrast, the PPAR-α agonist yielded markedly higher *PPAR-α* gene expression in the C-α group (+255%, P<0.0001, Fig 5A) and in the HF-α group (+1305%, P<0.0001, Fig 5A) in comparison to its counterparts. Two-way ANOVA explained these observations as diet significantly influenced the *PPAR-α* gene levels (P = 0.0402), but only PPAR-α treatment influenced this parameter, being the most potent stimulus, accounting for 76% of its total variance.

Conversely, *PPAR-β/δ* gene expression was enhanced by both treatments. The treatment with the PPAR-α agonist yielded higher *PPAR-β/δ* gene expression in C-α group than in the C group (+174%, P<0.0001, Fig 5B) and in HF-α group than in the HF group (4417%, P<0.0001). Besides, the treatment with the PPAR-β/δ agonist led to higher *PPAR-β/δ* gene expression in C-β (+215%, P<0.0001, Fig 5B) and in HF-β (+4369%, P<0.0001, Fig 5B) than in its counterparts. Two-way ANOVA revealed that PPAR-α treatment and PPAR-β/δ treatment, independently, influenced the *PPAR-β/δ* gene levels (P<0.0001). Also, both treatments interacted with the diet to influence *PPAR-β/δ* gene levels (P = 0.0012 for the PPAR-α agonist and P = 0.0192 for the PPAR-β/δ agonist).

PPAR-γ gene expression was equally increased by both treatments. The C-α and C-β groups presented with a higher *PPAR-γ* gene expression than the C group (+1228%, P<0.0001 and +1073%, P = 0.0005 Fig 5C), as well as HF-α and HF-β showed raised PPAR-γ gene expression when compared to the HF group (+10964%, +10030% and P<0.0001 Fig 5C). Diet interacted with both treatments to determine the PPAR-gamma gene expression (Two-way ANOVA, P<0.001. There was no difference between PPAR-α or the PPAR-β/δ treatment on this parameter as both accounted for 53% of PPAR-gamma gene levels total variance (Two-way ANOVA, P<0.0001).

The increased gene expression of all PPAR isoforms after the treatment with the PPAR-α agonist was directly related to the increase in *PGC1-α* gene expression in C-α and HF-α groups when compared with their counterparts (+198%; P = 0.0009 and +17844%; P<0.05, Fig 5D). Only the PPAR-α treatment influenced the *PGC1-α* gene levels, accounting for 66% of its total variance (Two-way ANOVA, P<0.0001).

**Fig 6:**
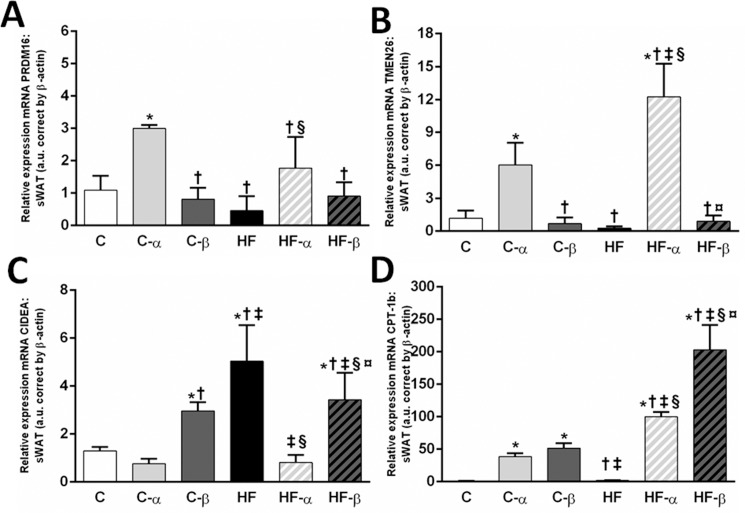
**sWAT gene expressions of *PRDM16* (A), *TMEM26* (B), CIDEA (C) and *CPT1-b* (D).** Beta-actin was used as an internal control to normalize selected gene expression. Values are the means ± SD, n = 5. Differences between the groups are indicated: P<0.05 when compared with the C group [*], C-α group [†], C-β group [‡], HF group [§] and HF-α [¤] (one-way ANOVA and post-hoc test of Holm-Sidak). Groups: C (control group), α (PPAR-α agonist WY14643), β (PPAR-β/δ agonist GW0742), HF (High-fat diet group).

The *PRDM16*, essential to the maintenance of the beige phenotype in white adipocytes, was increased only in the groups treated with the PPAR-α agonist. The C-α group showed higher *PRDM16* gene levels than the C group as well as the HF-α group exhibited higher *PRDM16* gene levels than the HF group (+174% and +287%, P<0.005, Fig 6A), suggesting the presence of viable beige cells in the sWAT after PPAR-α treatment. Two-way ANOVA confirmed these findings as only the PPAR-α treatment influenced this parameter, accounting for 56% of the *PRDM16* total variance (P<0.0001, Fig 6A).

The PPAR-α agonist exclusively influenced the *TMEM-26* gene expression in both treated groups. The C-α group presented with higher TMEM-26 gene expression than the C group (+412%, P<0.0001, Fig 6B), as well as the HF-α group, showed higher *TMEM-26* gene levels than the HF group (+4486%, P<0.0001, Fig 6B). Two-way ANOVA results showed that only the PPAR-α treatment influenced the TMEM-26 gene levels significantly, accounting for 53% of its total variance (P<0.0001).

On the contrary, the *CIDEA* gene expression was more influenced by the PPAR-β/δ agonist treatment. The C-β group presented with higher CIDEA gene expression than the C group (+128%, P = 0.0027, Fig 6C), whereas both HF treated groups showed reduced *CIDEA* gene levels than the HF group. However, the HF-α presented with a more pronounced reduction (-84%, P<0.0001, Fig 6C) than the HF-β (-32%, P<0.0343, Fig 6C) when compared to the untreated HF group. Two-way ANOVA showed that the diet influenced the CIDEA gene levels and interacted with both treatments significantly (P = 0.0015). However, only the PPAR-α treatment was able to affect the CIDEA gene expression as a single stimulus, accounting for 39% of its total variance (P<0.0001).

*CPT-1b* gene expression was also increased in C-α and C-β when compared to C group (+3541% P = 0.0022 and +4781% P = 0.0003, Fig 6D) and in the HF-α and HF-β when compared to the HF group (+4.945% and +10.173%, P<0.0001, Fig 6D). Notably, the PPAR-β/δ agonist yielded a higher *CPT-1b* gene expression than the PPAR-α agonist in animals fed with the HF diet (HF-β: +104% compared to HF-α, Fig 6D). Two-way ANOVA showed a significant interaction between diet and both treatments regarding the *CPT-1b* gene levels (P<0.0001).

**Fig 7:**
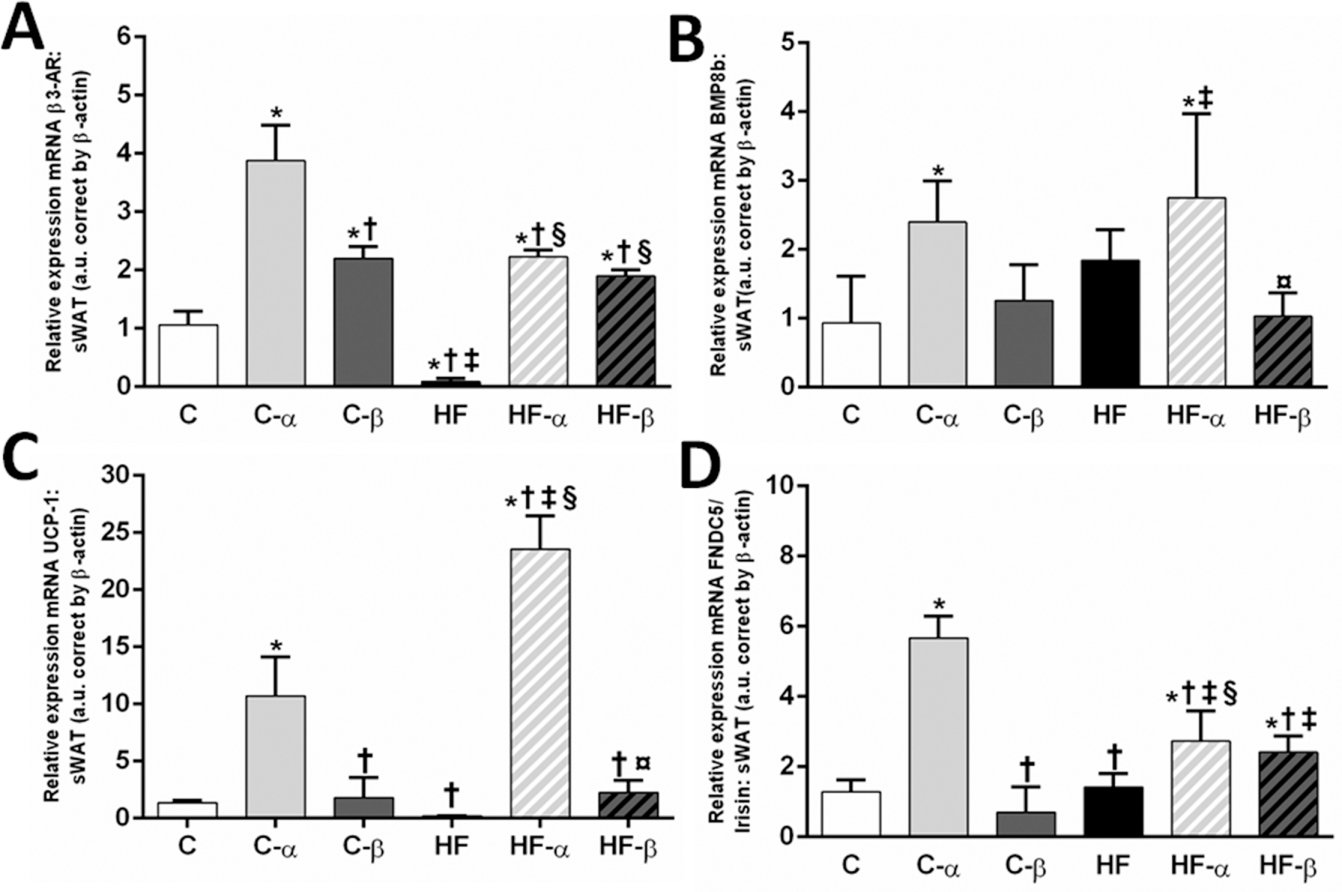
**sWAT gene expressions of *β3-AR* (A), *BMP8b* (B), *FNDC5/Irisin* (C) and *UCP1* (D).** Beta-actin was used as an internal control to normalize selected gene expression. Values are the means ± SD, n = 5. Differences between the groups are indicated: P<0.05 when compared with the C group [*], C-α group [†], C-β group [‡], HF group [§] and HF-α [¤] (one-way ANOVA and post-hoc test of Holm-Sidak). Groups: C (control group), α (PPAR-α agonist WY14643), β (PPAR-β/δ agonist GW0742), HF (High-fat diet group).

Both treatments elevated the gene expression of *β3-AR*. The C-α and the C-β groups presented with higher *β3-AR* gene levels than the C group (+265%, P<0.0001 and +107%, P = 0.0012, respectively, Fig 7A). Likewise, the HF-α and HF-β groups showed higher *β3-AR* gene expression than the HF group (+2456%, + 2081% P<0.0001, Fig 7A). Diet and both treatments exerted a significant influence on *β3-AR* gene levels (Two-way ANOVA, P<0.0001). However, only the PPAR-α treatment interacted significantly with the diet to influence *β3-AR* gene levels (Two-way ANOVA, P = 0.0371).

On the contrary, *BMP8b* gene, associated with the acute thermogenic signaling and typically expressed in BAT, was increased just in the groups treated with the PPAR-α agonist. The C-α group showed higher *BMP8b* gene levels than the C group as well as the HF-α group exhibited higher *BMP8b* gene levels than the HF group (+157% and +72% + P<0.05, respectively, Fig 7B), indicating the presence of brown-like adipocytes (Fig 7B). PPAR-α treatment exerted the primary influence on *BMP8b* gene levels, accounting for 47% of its total variance (Two-way ANOVA, P = 0.0003).

Along with the β3-AR and *BMP8b* gene expressions, the *FNDC5/Irisin* gene was enhanced in the C-α and HF-α groups when compared with their counterparts (+340%, P<0.0001 and +92% P<0.05, respectively, Fig 7C). Importantly, the treatment with the PPAR-β/δ agonist did not change its levels significantly. Two-way ANOVA highlighted the primary effect of PPAR-α treatment, which accounted for 60% of its total variance, while the diet accounted for 15% and the interaction between diet and treatment accounted for 18% of the *FNDC5/irisin* total variance.

FNDC5/irisin results agreed with the increased *UCP1* gene expression in the C-α and HF-α groups when compared with their counterparts (+687% and +1045% P<0.0001, respectively, Fig 7D). Much as the PPAR-β/δ treatment restored the *UCP1* gene levels in the HF-β group, showing values similar to C group, it was much lower than the HF-α (-90%, P<0.0001). Two-way ANOVA showed that the PPAR-α treatment accounted for 73% of the *UCP1* total variance (P<0.0001), whereas the PPAR-β/δ treatment accounted for only 28% (P = 0.0150). Importantly, there was a significant interaction between diet and PPAR-α treatment regarding *UCP1* gene expression (P<0.0001).

**Fig 8:**
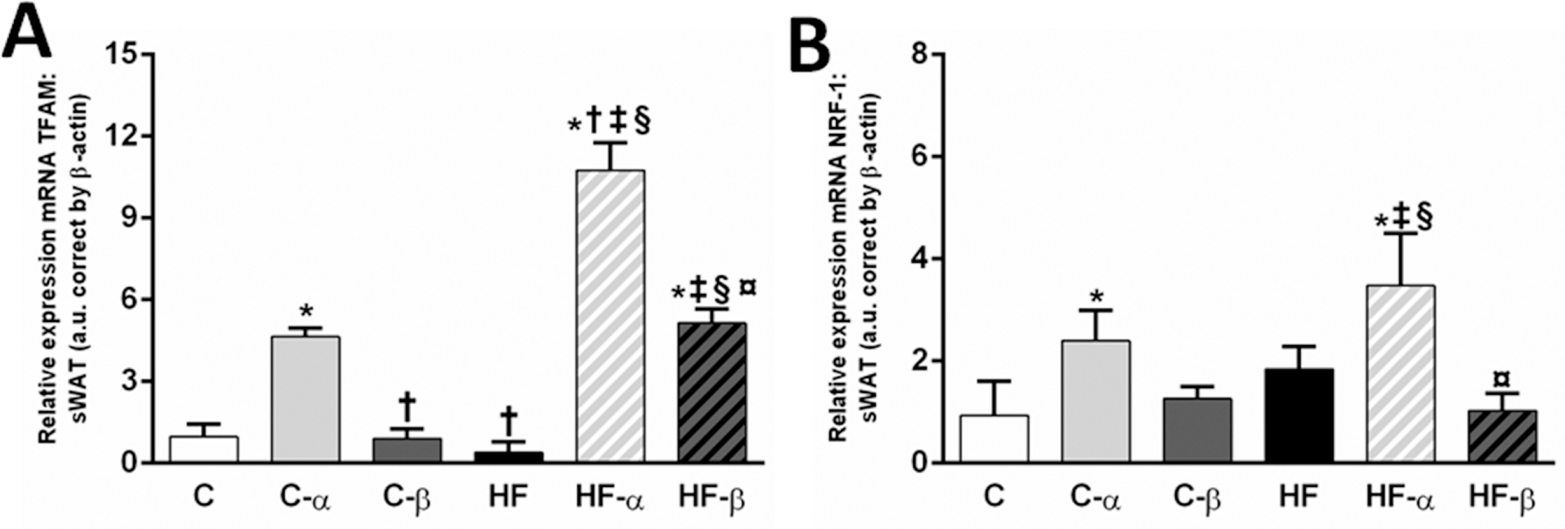
**sWAT gene expressions of *TFAM* (A) and *NRF-1* (B).** Beta-actin was used as an internal control to normalize selected gene expression. Values are the means ± SD, n = 5. Differences between the groups are indicated: P<0.05 when compared with the C group [*], C-α group [†], C-β group [‡], HF group [§] and HF-α [¤] (one-way ANOVA and post-hoc test of Holm-Sidak). Groups: C (control group), α (PPAR-α agonist WY14643), β (PPAR-β/δ agonist GW0742), HF (High-fat diet group).

*TFAM* and *NRF-1*, which are crucial genes to mitochondrial biogenesis, were altered after the treatments. The C-α group presented with higher *TFAM* gene expression than the C group (+377%, P<0.0001, Fig 8A), whereas both the HF-α and the HF-β groups, showed higher *TFAM* gene levels than the HF group (+2619% and +1199%, P<0.0001, Fig 8A). Two-way ANOVA revealed that diet and both treatments influenced independently the *TFAM* gene levels (P<0.0001), besides interacting significantly to determine this parameter (P<0.0001). However, the PPAR-α treatment, independently, accounted for 71% of *TFAM* gene levels total variance, exerting the main effect (P<0.0001).

In contrast, *NRF-1* gene expression was only influenced by the PPAR-α agonist in both treated groups. The C-α group presented with higher *NRF-1* gene expression than the C group (+635%, P<0.005, Fig 8B) as well the HF-α group showed higher *NRF-1* gene levels than the HF group (+431%, P = 0.007, Fig 8B). Both diet and PPAR-α treatment showed an action as a single stimulus (P<0.0001). However, the main effect stemmed from the PPAR-α treatment, which accounted for 48% of the *NRF-1* gene expression total variance.

## Discussion

Our study identified the PPAR-α agonist WY14643 as a likely inducer of beige adipocyte formation in the sWAT, while the PPAR-β/δ agonist GW0742 improved metabolic parameters and stimulated the beta-oxidation, albeit with less effect on the browning phenomenon and the associated adaptive thermogenesis. The current findings indicate that obese animals treated with the PPAR-α or the PPAR-β/δ agonist benefited from weight loss, enhanced insulin sensitivity, and standard adipocyte size. Nevertheless, enhanced gene expression of markers from brown/beige adipocytes in the sWAT (*UCP1*, *TEMEM26*, *BMP8B*, and *PRDM16*) was noticeable only after the treatment with the PPAR-α agonist, suggesting active adaptive thermogenesis in this site. These observations comply with the presence of UCP1 positive beige adipocytes in the sWAT, which confirmed the browning phenomenon associated with the PPAR-α treatment.

About BM, both PPAR-α and PPAR-β/δ treatments yielded a noticeable weight loss, as previously described in the literature [16–18]. Notably, the treated groups had no difference in energy intake when compared to the respective untreated group, reinforcing that the reduced BM was a direct result of the PPARs activation and discarded the need for a pair-feeding group.

Reduced BM in both treated groups agrees with the reduced adiposity index and the consequent improvement in insulin resistance [19–22]. Adiposity and insulin resistance are highly influenced by PPARs expression. Rodents present low basal PPAR-α expression and moderate PPAR-β/δ expression in WAT [23–25] and their pharmacological induction has been regarded as promising tools to tackle metabolic constraints stemmed from obesity [25]. In this context, PPAR-α activation has been associated to enhanced EE due to the stimulation of adaptive thermogenesis in the sWAT and BAT [12, 26]. Also, reduced adipocyte size due to PPAR-α or PPAR-β/δ activation is correlated with the ability of these PPAR isoforms to induce β-oxidation, shown herein by the enhanced *CPT-1b* gene levels due to both treatments [27, 28]. PPAR-α activation seems to trigger β-oxidation by augmenting the mitochondrial input of fatty acids for metabolism [29, 30]. In contrast, PPAR-β/δ activation is linked to a change in the preference for lipid as the primary cellular fuel instead of carbohydrate, which ameliorates the glucose-stimulated insulin secretion and promotes increased insulin sensitivity in the peripheral tissues [31, 32].

Reduced fat deposits are closely related to the increased number of small adipocytes in contrast to the reduced number of the hypertrophied ones [12, 33]. Enlarged adipocytes in the HF group comply with the hyperinsulinemia found because of the disrupted adipoinsular axis, caused by the chronic HF diet intake [34]. These alterations encompass hyperleptinemia, sustained by the augmented adipocyte size, and the lost capacity of leptin to inhibit the postprandial insulin secretion by the pancreas [35]. Hence, the hyperinsulinemia compromises the pancreatic islet function in the long run, besides stimulating lipogenesis and, thus, adipocyte hypertrophy, while inhibiting adipocyte hyperplasia and its white/brown plasticity [35, 36].

Conversely, both treatments normalized the adipocyte size and the insulin levels, which were previously shown due to PPAR-α, PPAR-β/δ, and pan-PPAR activation [12, 21, 27]. Adipocytes with normal size preserve its hyperplasia capacity [36], which prevents insulin resistance, maintains the adipokine release towards a good profile and allows its multilocularization to acquire a brown phenotype [37]. FNDC5/irisin seems to act in an autocrine/paracrine fashion, as an adipokine, to stimulate browning once its skeletal muscle secretion correlates with insulin resistance in diet-induced obesity [38], but does not influence the adipocyte plasticity towards a thermogenic phenotype [20]. Body mass loss and reduced adiposity are often followed by a reduced FNDC5/irisin expression in the sWAT, which seems to crosstalk with the skeletal muscle in a feedback mechanism [39, 40]. PPAR-α treated animals showed reduced adiposity with increased local FNDC5/irisin gene expression and circulating levels, which might suggest a role for the PPAR-α activation in the adipose tissue-skeletal muscle axis.

As for thermogenic pathway, the β3-AR acts as an initiator [41]. It is clear that without the sympathetic stimulation, there is no adaptive thermogenesis and, therefore, the beige adipocytes rely on an adequate coupling between the β3-AR and the UCP1 activity (the thermogenesis effector) to enhance EE by consuming the chemical energy and producing heat, instead of generating ATP [6]. It should be said that both treatments resulted in raising *β3-AR* gene levels. However, only the PPAR-α treatment enhanced *BMP8b* gene levels, which acts centrally to enhance the sympathetic output to the thermogenic site and locally to amplify the thermogenic response to the adrenergic stimulus [42]. In this regard, knockout BMP8b animals become more obese than wild animals when fed an HF diet, while the ablation of PPAR-α also turns rodents more prone to obesity due to the reduced ability to use lipids as fuel to the thermogenesis [42, 43]. These observations put forward a role for PPAR-α in the coupling between *β3-AR* and *UCP1* through *BMP-8b*.

It should be noted that PPAR-β/δ treated animals did not show enhanced *PRDM16* gene levels. *PRDM16* is an essential gene to the brown adipocyte lineage differentiation, while it is not crucial to the mature brown adipocyte maintenance [44]. On the other hand, the beige adipocytes rely on enhanced *PRDM16* expression to keep its thermogenic phenotype. As browning is a reversible phenomenon, the beige adipocyte turns into a white one if the *PRDM16* gene levels are not enough to sustain its multilocular phenotype [45]. So, it can be argued that the steady *PRDM16* gene levels help to explain the predominant unilocular form of the adipocytes from the PPAR-β/δ treated animals.

PPAR-α has *UCP1* as a target gene [46]. UCP1 is placed in the inner mitochondrial membrane and acts as an alternative channel to H^+^ protons return from the intermembrane space and, thus, preventing from the ATP synthesis and allowing the resulting energy to be released as heat [11, 47]. The presence of the highest body temperatures among all the groups coupled with the positive UCP1 labeling, high PRDM16, UCP1 and TMEM26 gene expression in the PPAR-α treated groups confirm the presence of metabolically active beige cells [48] and comply with the observation of multilocular adipocytes in these animals.

TMEM26 gene was exclusively induced by the PPAR-α treatment and confirms the beige phenotype as the beige adipocytes much more express it than by the brown or white adipocytes [49]. Also, PPAR-α seems to act via FNDC5/irisin to enhance the UCP1 performance [50], maximizing the capacity of these new beige adipocytes to produce heat instead of accumulating ATP.

The *CIDEA* expression might explain the differences regarding *UCP1* gene expression and protein labeling between animals submitted to the two treatments. CIDEA and UCP1 are both localized to the mitochondria, and CIDEA was identified as a likely inhibitor of the UCP1 activity by forming a complex with it [51], besides being positively related to the size of lipid droplet (LD) [52]. Hence, the PPAR-β/δ treated animals showed enhanced *CIDEA* gene expression coupled with large LD within their adipocytes, which was accompanied by a reduced UCP1 gene expression and negative UCP1 cells under the confocal microscopy evaluation.

On the other hand, it is reasonable to say that the reduced CIDEA expression by the PPAR-α treatment complied with the high UCP1 gene expression and UCP1 protein labeling by immunofluorescence. Moreover, reduced CIDEA expression favors the multilocularization of adipocytes by enhancing lipolysis rate, whereas CPT-1b enhances the beta-oxidation. Both events guarantee the substrate supply to the enhanced thermogenesis, leading to a lean phenotype [51, 53].

As for the multilocularization process, mitochondrial biogenesis plays a pivotal role to sustain this new cell physiology, headed for thermogenesis instead of lipogenesis and lipid storage [54]. The coupling between the nucleus and mitochondria is made by the NRF-1, which signals to the TFAM to activate the mitochondrial DNA duplication [55]. The PPAR-α treatment enhanced both *NRF-1* and *TFAM* gene levels.

Much as the PPAR-β/δ treatment did not cause relevant thermogenesis on the basis that a possible CIDEA and UCP1 interaction occurred. HF-β animals showed the highest *CPT-1b* gene expression, which is linked to the enhanced use of lipids as fuel by the adipocytes [56] and skeletal muscle [57] and may be associated to the reduced adipocyte size, the normalized glucose tolerance and the lean phenotype [32].

It is worth mentioning that the higher sWAT PPAR-γ expression observed after both treatments, irrespective of the diet, did not seem to influence the browning phenomenon herein since only PPAR-γ full agonists elicit browning [58].

**Fig 9:**
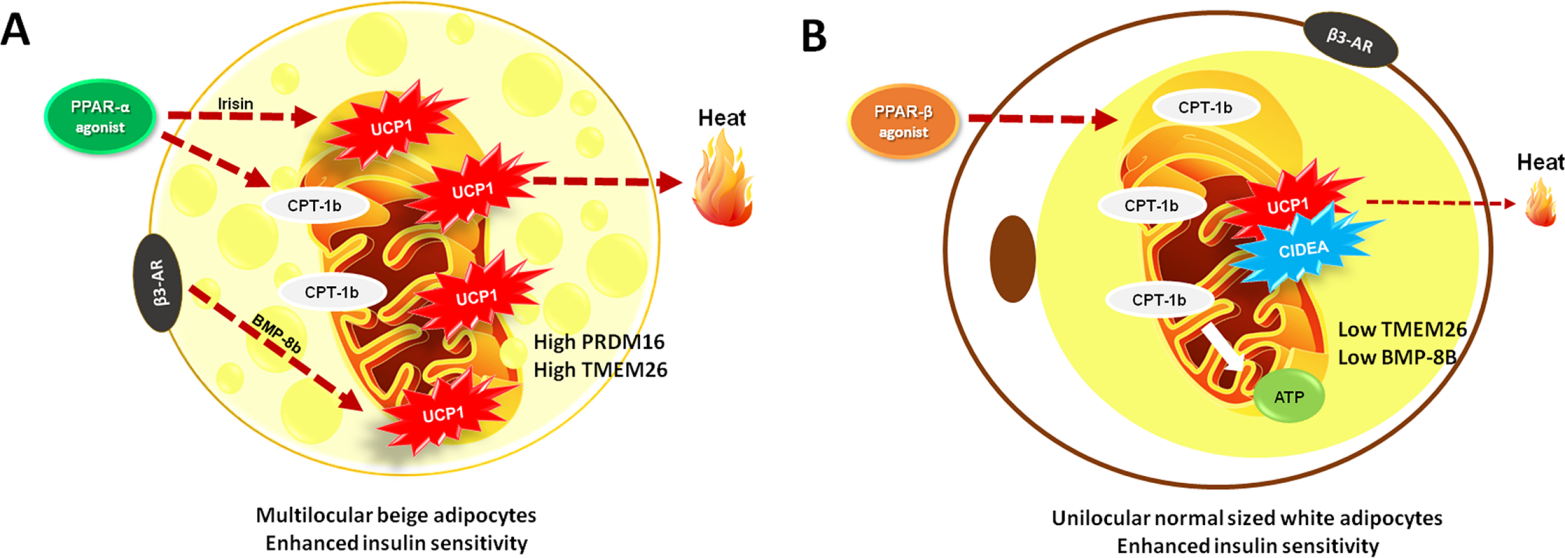
Scheme of the primary results obtained in the study. A) Animals treated with the PPAR-α agonist WY-14643 presented with beige adipocytes predominantly in the sWAT, characterized by high *PRDM16*, *TMEM26* and *UCP1* gene expressions, UCP1 protein labeling and high body temperature. Substrates that sustained enhanced thermogenesis stemmed from the enhanced beta-oxidation coupled with the high adrenergic output indicated by the elevated BMP-8b gene levels. B) Animals treated with the PPAR-β/δ agonist GW0742 presented with normal sized white adipocytes predominantly in the sWAT, characterized by low *TMEM26*, BMP-8b, and steady *PRDM16* gene levels when compared to the untreated HF. Beneficial metabolic effects were caused by the marked beta-oxidation, which reduced the adipocyte size and ameliorated the insulin sensitivity. Notably, the increased CIDEA expression and its capacity to inhibit the UCP1 activity may explain the less evident adaptive thermogenesis.

Some central concluding remarks are that the GW0742 increased *PPAR-β/δ* gene expression, without changing the PPAR-α gene levels, thus isolating the results of the PPAR-β/δ isoform, which was less potent to induce thermogenesis in the sWAT. We propose that a possible interaction between CIDEA and UCP1 occurs when *PPAR-β/δ* is activated without a *PPAR-α* co-activation. Also, it should be highlighted that that the temperature of 21°C, used in the present study, does not affect the UCP1 expression in the sWAT neither can trigger itself the browning phenomenon. Only the temperature of 4°C can trigger browning, discarding the use of a pharmacological inducer [59]. Hence, we can conclude that the browning phenomenon observed herein can be entirely attributed to the WY14643 agent. Fig 9 summarizes the primary type of adipocyte found in the sWAT after the PPAR-α and PPAR-β/δ treatments and its physiology.

## Conclusions

Overall, our results point to a decisive role of PPAR-α agonism to the browning of the sWAT, whereas the PPAR-β/δ agonism was less likely to activate the thermogenic pathway in this non-thermogenic site probably due to the enhanced CIDEA expression. Even though beneficial metabolic effects were driven by both PPAR agonists and involved enhanced mitochondrial beta-oxidation, reduced adipocyte size, and increased insulin sensitivity, only the PPAR-α treatment enhanced the *TMEM26* and *PRDM16* gene levels, besides yielding positive UCP1 labeling in multilocular beige adipocytes coupled with high body temperature. These observations comply with increased adaptive thermogenesis and put forward a possible translational potential for the PPAR-α agonists when it comes to obesity management.
